# Sex hormones and risk of epilepsy: A bidirectional Mendelian randomization study

**DOI:** 10.3389/fnmol.2023.1153907

**Published:** 2023-04-11

**Authors:** Bin Ke, Chunyu Li, Huifang Shang

**Affiliations:** Laboratory of Neurodegenerative Disorders, Department of Neurology, National Clinical Research Center for Geriatrics, West China Hospital, Sichuan University, Chengdu, Sichuan, China

**Keywords:** hormone, epilepsy, Mendelian randomization, causation, estradiol

## Abstract

**Background:**

Multiple evidence has suggested complex interaction between sex hormones and epilepsy. However, whether there exists a causal association and the effect direction remains controversial. Here we aimed to examine the causative role of hormones in the risk of epilepsy and vice versa.

**Methods:**

We conducted a bidirectional two-sample Mendelian randomization analysis using summary statistics from genome-wide association studies of major sex hormones including testosterone (*N* = 425,097), estradiol (*N* = 311,675) and progesterone (*N* = 2,619), together with epilepsy (*N* = 44,889). We further performed sex-stratified analysis, and verified the significant results using summary statistics from another study on estradiol in males (*N* = 206,927).

**Results:**

Genetically determined higher estradiol was associated with a reduced risk of epilepsy (OR: 0.90, 95% CI: 0.83–0.98, *P* = 9.51E-03). In the sex-stratified analysis, the protective effect was detected in males (OR: 0.92, 95% CI: 0.88–0.97, *P* = 9.18E-04), but not in females. Such association was further verified in the replication stage (OR: 0.44, 95% CI: 0.23–0.87, *P* = 0.017). In contrast, no association was identified between testosterone, progesterone and the risk of epilepsy. In the opposite direction, epilepsy was not causally associated with sex hormones.

**Conclusion:**

These results demonstrated higher estradiol could reduce the risk of epilepsy, especially in males. Future development of preventive or therapeutic interventions in clinical trials could attach importance to this.

## Introduction

Epilepsy is one of the most common and disabling neurologic conditions characterized by recurrent seizures caused by aberrant neuronal networks in the brain (Thijs et al., 2019). The detailed pathophysiology of epilepsy was still poorly understood, and multiple clinical and lifestyle factors could increase the risk of seizures like head injury, stress, alcohol use or drug abuse. Identifying risk factors for epilepsy could help better understand the disease pathogenesis, and provide care and therapeutic strategies for patients and clinicians.

Sex hormones, which could influence brain excitability, were suggested to be involved in various ways in the manifestations of epilepsy (Harden and Pennell, 2013). Sex hormones, mainly including estrogens, androgens and progestogens, are molecules produced by the endocrine system that send messages to various parts of the body, and help regulate the body’s processes. There is a complex, bidirectional interaction between sex steroid hormones and epilepsy, with hormones affecting the incidence and frequency of seizures, and vice versa. For example, there have been reports of correlation between increasing serum testosterone levels and a sustained reduction in seizure frequency and intensity (Heiry et al., 2015). Progesterone combined with antiepileptic therapy was suggested to result in a significant reduction of seizure frequency in the patients with catamenial epilepsy based on the treatment results on 36 women aged 20–40 years with seizures (Motta et al., 2013). Meanwhile, several studies about chronic estrogen administration in patients or animal model showed association between estrogen and epilepsy, though whether estrogen was anticonvulsant or proconvulsant or non-relevant was still incongruent (Velísková et al., 2010). In addition, most antiepileptic drugs interact with both epilepsy itself and hormones. These multiple evidence suggested the essential role of hormone in the pathogenesis of epilepsy. In contrast, a prospective study among 114,847 Nurses’ Health Study II participants followed from 1989–2005 identified no significant association between any reproductive factor and risk of unprovoked seizure (Dworetzky et al., 2012). However, the observational studies might be biased by unavoidable confounding factors and limited sample size, and cannot determine causation. Therefore, the causal association between hormones and epilepsy is still elusive and needs further exploration.

In this context, we performed a bidirectional two-sample Mendelian randomization (MR) analysis to explore the causal role of hormones including estrogens measured by estradiol, androgens measured by testosterone, and progestogens in the risk of epilepsy, and vice versa. MR analysis is an analytical method that uses genetic variants which are fixed at conception as instrumental variables for modifiable risk factors that affect an outcome (Davies et al., 2018). It is increasingly being used because it can overcome a major limitation of evidence from observational studies: unmeasured confounding (Li et al., 2021, 2022). The MR approach is less susceptible to reverse causation or confounding factors which may distort the interpretations of conventional observational studies. As a result, we found that higher estradiol was causally associated with a reduced risk of epilepsy, particularly in males.

## Methods

### Datasets

We obtained summary statistics of total testosterone (*N* = 425,097) from a previous large genome-wide association study (GWAS) based on genotype and phenotype data from the UK Biobank (Ruth et al., 2020). Testosterone was measured by “one step competitive analysis on a Beckman Coulter Unicel Dxl 800”. Summary statistics of estradiol were from another GWAS based on data from the UK Biobank (Schmitz et al., 2021). Estradiol was measured by “two step competitive analysis on a Beckman Coulter Unicel Dxl 800”. Estradiol in both sexes was obtained with meta-analysis of estradiol in males and females using metal (*N* = 311,675). Summary statistics of progesterone (*N* = 2,619), progesterone in males (*N* = 1,358), and progesterone in females (*N* = 1,261) were from GWAS on steroid hormone levels based on individuals of European ancestry (Pott et al., 2019). Progesterone was measured by liquid chromatography-tandem mass spectrometry. Details of the summary data from all GWAS were listed in Supplementary Table 1. As a replication, we further validated the results using summary statistics from another GWAS of estradiol in males (*N* = 206,927) (Ruth et al., 2020). This study analyzed estradiol levels as a binary phenotype, and included samples with low estradiol levels. Therefore, though there is some sample overlap, the study in the replication stage had larger sample size and utilized different analysis workflow from the study in the discovery stage. Single nucleotide polymorphisms (SNP) that passed the genome-wide significance threshold (*P* < 5E-08) were chosen as instrumental variants, which were then clumped based on the 1,000 Genomes Project linkage disequilibrium (LD) structure. Index SNPs (R2 < 0.001 with any other associated SNP within 10 Mb) with the minimum *P* value were kept.

We obtained GWAS summary statistics of epilepsy from a genome-wide meta-analysis (*N*_*case*_ = 15,212, *N*_*control*_ = 29,677) (The International League Against Epilepsy Consortium on Complex Epilepsies, 2018). Seizures and epilepsy syndromes were diagnosed according to the classification and terminology outlined by the International League Against Epilepsy. Harmonization was undertaken to rule out strand mismatches and ensure alignment of SNP effect sizes.

### Mendelian randomization analysis

We hypothesized that sex hormones as a risk factor could causally influence the risk of epilepsy, and the following assumptions were satisfied: the genetic variants used as instrumental variables are associated with sex hormone levels; the genetic variants are not associated with any confounders; the genetic variants are associated with risk of epilepsy through sex hormones (namely horizontal pleiotropy should not be present).

To evaluate the causative effect of sex hormones on the risk of epilepsy, we performed a two-sample MR analysis using the random effects inverse variance weighted (IVW) method, which is most widely used in MR studies and could provide robust causal estimates under the absence of directional pleiotropy (Supplementary Figure 1). A *P* value below 0.017 (0.05/3) was considered statistically significant after the Bonferroni correction. For the significant association, we further verified the results using the weighted median method, which generally has greater power with a positive causal effect, particularly as the proportion of invalid instrumental variables increases.

In the second stage, we evaluated whether epilepsy as a risk factor could causally influence the amount of sex hormones, and performed the MR analysis using the same workflow. Given that only three loci were significant for epilepsy, a more relaxed significance threshold (*P* < 5E-07) was used. Since the full summary statistics of GWAS on progesterone were not available, we did not analyze progesterone as an outcome in the opposite direction. In addition, we conducted comprehensive sensitivity analyses to estimate potential violations of the model assumptions in the MR analysis. We conducted Mendelian randomization pleiotropy residual sum and outlier (MR-PRESSO) analysis to detect outlier instrumental variables, which were removed step-by-step to reduce the effect of horizontal pleiotropy. Cochran’s Q test was executed to check the heterogeneity across the individual causal effects. MR-Egger regression was performed to evaluate the directional pleiotropy of instrumental variables. To evaluate the strength of each instrumental variable, we computed the F-statistic of each SNP. The statistical analyses were conducted using the R package TwoSampleMR 0.5.5.

## Results

**FIGURE 1:**
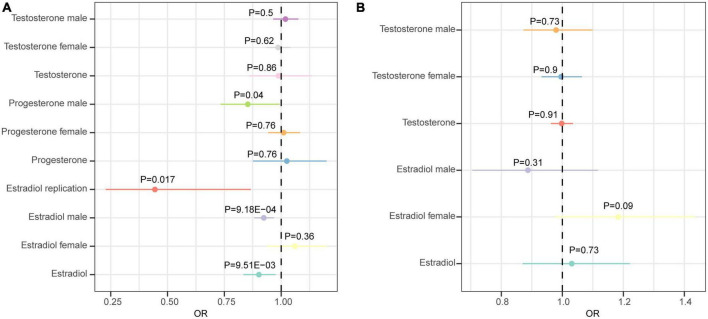
Forest plot showing results from the Mendelian randomization analysis. **(A)** Results from the Mendelian randomization (MR) analysis to evaluate the causal role of hormone traits in epilepsy using the inverse variance weighted method. **(B)** Results from the MR analysis to evaluate the causal role of epilepsy in hormone traits using the inverse variance weighted method. Estimates are per 1 standard deviation (SD) increase in the trait.

**FIGURE 2:**
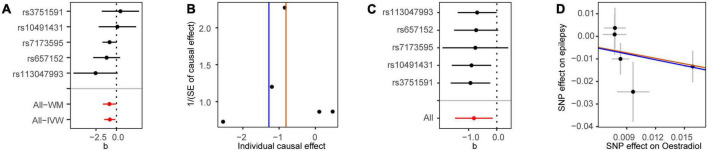
Mendelian randomization analysis results for estradiol in males on the risk of epilepsy. **(A)** Forest plot of the causal effect of estradiol in males on epilepsy. **(B)** Funnel plot showing the estimation using the inverse of the standard error of the causal estimate with each individual SNP as a tool. The vertical line represents the estimated causal effect. SNP, single nucleotide polymorphism. **(C)** Forest plot of the results of the leave-one-out sensitivity analysis, where each SNP was iteratively removed from the instrumental variables. **(D)** Scatter plot of SNP effects on estradiol in males and epilepsy. The 95% CI for the effect size on epilepsy is shown as vertical lines, while the 95% CI for the effect size on estradiol in males is shown as horizontal lines. The slope of fitted lines represents the estimated MR effect per method.

**FIGURE 3:**
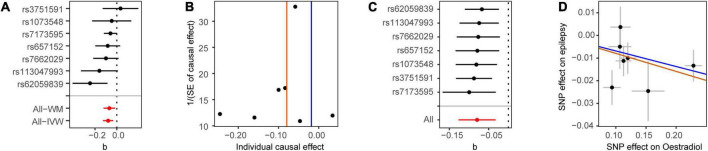
Mendelian randomization analysis results for estradiol in males on the risk of epilepsy in the replication stage. **(A)** Forest plot of the causal effect of estradiol in males on epilepsy. **(B)** Funnel plot showing the estimation using the inverse of the standard error of the causal estimate with each individual SNP as a tool. The vertical line represents the estimated causal effect. SNP, single nucleotide polymorphism. **(C)** Forest plot of the results of the leave-one-out sensitivity analysis, where each SNP was iteratively removed from the instrumental variables. **(D)** Scatter plot of SNP effects on estradiol in males and epilepsy. The 95% CI for the effect size on epilepsy is shown as vertical lines, while the 95% CI for the effect size on estradiol in males is shown as horizontal lines. The slope of fitted lines represents the estimated MR effect per method.

We first analyzed the role of each hormone in the risk of epilepsy. Results showed that higher estradiol was associated with a reduced risk of epilepsy using both the IVW (OR: 0.90, 95% CI: 0.83–0.98, *P* = 9.51E-03) and weighted mode methods (OR: 0.90, 95% CI: 0.82∼0.98, *P*: 0.014) (Figure 1A). The funnel plot displays a symmetric pattern of effect size variation around the point estimate (Figure 2). In the sex-stratified analysis, such a protective effect was detected in males using the IVW (OR: 0.92, 95% CI: 0.88–0.97, *P* = 9.18E-04) and weighted mode (OR: 0.93, 95% CI: 0.89–0.98, *P* = 0.01) methods, while in females no association was identified (Figure 1A and Supplementary Figures 2, 3). In contrast, no association was identified between testosterone, progesterone and epilepsy. Then we tried to validate the results using summary statistics from another GWAS on estradiol in males. As a result, higher estradiol was associated with reduced risk of epilepsy using both the IVW (OR: 0.44, 95% CI: 0.23∼0.87, *P*: 0.017) and weighted median (OR: 0.42, 95% CI: 0.19∼0.91, *P*: 0.029) methods. The funnel plot displays a symmetric pattern of effect size variation around the point estimate (Figure 3).

Next, we analyzed whether epilepsy as a risk factor could causally influence testosterone and estradiol levels using the MR approach. However, no significant association was identified (Figure 1B).

Furthermore, we performed extensive sensitivity analyses to validate the causal association between sex hormones and epilepsy. The Cochran’s Q test did not detect the heterogeneity of effects across the instrumental variables (Table 1). The F statistics of all the instrumental variables were above 10 (ranging from 24 to 1,656), indicating the absence of weakness in the selected instruments. No apparent horizontal pleiotropy was observed as the intercept of MR-Egger was not significantly deviated from zero. Meanwhile, no potential instrumental outlier was detected by the MR-PRESSO analysis. The leave-one-out results suggested the causal effect was not driven by a single instrumental variable.

**TABLE 1:** Heterogeneity and horizontal pleiotropy analyses between hormone traits and epilepsy.

Hormone traits	Heterogeneity			Horizontal pleiotropy			MR-PRESSO *P*-value
	IVW Q	IVW Q df	IVW *P*-value	Intercept	SE	*P*-value	
***Epilepsy as outcome***							
Testosterone	93.73	88	0.32	7.62E-04	2.24E-03	0.73	0.31
Testosterone in males	102.85	89	0.15	-1.98E-03	1.76E-03	0.26	0.15
Testosterone in females	132.69	112	0.09	4.11E-04	1.72E-03	0.81	0.09
Estradiol	1.40	3	0.70	-2.79E-03	0.01	0.87	0.80
Estradiol in males	7.60	6	0.27	-0.01	0.01	0.42	0.32
Estradiol in females	4.24	3	0.24	-0.02	0.02	0.42	0.25
Estradiol in males in replication	3.68	4	0.45	0.01	0.01	0.70	0.62
***Epilepsy as exposure***							
Testosterone	16.03	9	0.07	1.39E-03	2.65E-03	0.61	0.07
Testosterone in males	10.56	9	0.31	4.40E-04	0.01	0.94	0.31
Testosterone in females	10.86	9	0.29	-4.48E-03	4.45E-03	0.34	0.27
Estradiol	11.04	9	0.27	0.02	0.01	0.24	0.27
Estradiol in males	12.52	10	0.25	0.04	0.02	0.09	0.26
Estradiol in females	8.08	10	0.62	1.66E-03	0.02	0.93	0.66

IVW, inverse variance weighted; Q, Cochran’s Q test estimate; df, Cochran’s Q test degrees of freedom; SE, standard error.

## Discussion

In the current study, we investigated the causative role of three major hormones in the risk of epilepsy using the MR approach, and vice versa. The results showed that higher estradiol was associated with a reduced risk of epilepsy, especially in males. These findings provided a better understanding of the role of sex hormones in the risk of epilepsy, and had clinical implications for clinicians and researchers.

Though close correlation between estrogen and epilepsy has been reported, whether estrogen is proconvulsant or anticonvulsant remains controversial (Reibel et al., 2000; Taubøll et al., 2015). In the current study, we identified that genetically determined higher estradiol was associated with a reduced risk of epilepsy, especially in males. Similarly, one previous study found that estradiol facilitates the release of neuropeptide Y to suppress hippocampus-dependent seizures (Ledoux et al., 2009). Meanwhile, a previous *in vivo* study in male rats suggested estradiol treatment could both potentiate and attenuate kindled seizure in a time-dependent manner (Saberi et al., 2001). Estradiol could protect gamma aminobutyric acid metabolism and prevent spine synaptic loss in hippocampal slices in which epileptogenic activity is induced with bicuculine (Zhou et al., 2007). One study showed that docosahexaenoic acid (DHA) delays the onset of seizures by promoting the synthesis of 17β-estradiol in the brain, suggesting the anti-oxidative effects of 17β-estradiol may be involved in the prevention of seizures mediated by DHA (Ishihara et al., 2017). Meanwhile, estradiol replacement in ovariectomized female rats was shown to significantly reduce the seizure-related damage in the sensitive hilar region of hippocampal dentate gyrus (Iacobas et al., 2018), and estradiol administration to ovariectomized mice also exerted a significant neuroprotective effect against kainate-induced cell death (Schauwecker et al., 2009). These results suggested the potential protective role of estrogen in epilepsy. Notably, we did not detect such effect in females in the sex-stratified analysis, while one previous study has found the neuroprotective effects of chronic estradiol benzoate treatment on hippocampal cell loss induced by status epilepticus in the female rat as well (Reibel et al., 2000). Though estradiol might play various roles between sexes, we could not rule out the possibility that failure to detect association might be due to the limited power since the variance explained by the instrumental variables was relatively small (Supplementary Table 2). Therefore, future exploration based on summary data from GWAS with larger sample size was warranted to provide a more accurate estimate. Meanwhile, the protective effect of “higher” estrodiol in epilepsy also needs to be further quantified. Too high levels of estradiol might have a damaging effect as well. However, we could not evaluate whether there exists a U-shaped effect of estrodiol on epilepsy based on current datasets. Further studies investigating hormones in epilepsy could pay attention to the effect of excessive levels of estrodiol. In addition, the results should be interpreted with caution since the effect of sex hormones is very complex regulated by the hypothalamic-pituitary-gonadal axis, and the hormone level as a single factor could not model the real effect of sex hormoens in the body. Besides sex, age was also an important factor in determining the effect of sex hormones in epilepsy. Further exploration targeting patients in different age groups was still necessary.

Among the instrumental variables of estradiol, two (rs3751591, rs7173595) were in the gene *CYP19A1*, which encodes the aromatase cytochrome P450 (P450arom) enzyme, or estrogen synthase. The estrogen synthase converts androgens to estrogens, and previous experimental studies in laboratory animals have indicated a prominent role of brain aromatization of androgens to estrogens in regulating brain functions (Azcoitia et al., 2021). Notably, the epileptogenic effect of estradiol has also been reported. In males, reduced testosterone metabolite 3α-androstanediol decreases the frequency of seizures, whereas the aromatization of testosterone to estradiol increases seizures (Reddy, 2004). These paradoxical effects of estradiol could be explained by the rapid synaptic actions of the hormone, which potentiate excitatory transmission and suppress inhibition in neurons (Huang and Woolley, 2012), together with the long-term transcriptional hormonal effects, which decrease excitotoxicity and oxidative stress, inhibit apoptosis, and promote synaptogenesis (Azcoitia et al., 2019). Therefore, the role of estradiol in epilepsy might be related to the aromatase activity in the brain. Assess the potential neurological long-term effects of aromatase inhibitors when considering their potential clinical use for the treatment of epilepsy might provide novel insights.

Testosterone has a mixed effect on brain excitability and seizure threshold, with the estradiol metabolite of testosterone increasing brain excitability, while the reduced metabolite of testosterone, 3α-androstanediol, decreasing brain excitability. The complex correlation between testosterone and epilepsy has been documented a lot. For example, free testosterone concentrations were significantly lower in patients with epilepsy compared with healthy men, and successful temporal lobe epilepsy surgery led to a normalization of serum androgen concentrations in patients with complete seizure control (Bauer et al., 2000). Similarly, long-term EEG monitoring established a correlation between increasing serum testosterone levels and a sustained reduction in seizure frequency and intensity in a case with epilepsy (Heiry et al., 2015). The proconvulsant effects of testosterone have also been reported. The likely mechanism might involve aromatization of testosterone to estradiol, since the administration of aromatase inhibitors, which block the conversion of testosterone to estradiol, resulting in reduction of seizures (Harden and MacLusky, 2005). These multiple evidence suggested the close but still unclear correlation between testosterone and epilepsy, which needs further clarification. In the current study, from a genetic perspective using the MR approach, we did not identify significant association between testosterone and epilepsy. However, further exploration was still warranted.

Progesterone and its metabolites were mostly considered as anticonvulsant. Previous study suggested that treatment with progesterone reduces seizure frequency by more than half. However, in the current study we did not identify causal association between progesterone and epilepsy. This might be due to the limited effect of progesterone. Nevertheless, we cannot exclude the possibility that we failed to detect the association due to the insufficiency of current sample sizes as the effect might be relatively modest. Future studies on this topic are still needed.

In conclusion, our results demonstrated that higher estradiol was associated with a reduced risk of epilepsy. These findings help better understand the role of hormones in epilepsy, and will facilitate therapeutic management and drug discovery in future clinical trials.

## Data availability statement

The original contributions presented in this study are included in the article/Supplementary material, further inquiries can be directed to the corresponding authors.

## Author contributions

BK: conception and execution of research project, design, execution, review and critique of statistical analysis, and writing of the first draft of the manuscript. CL: execution of statistical analysis and review and critique of the manuscript. HS: organization of research project, review and critique of statistical analysis, and review and critique of the manuscript. All authors contributed to the article and approved the submitted version.
