# Supplementary material for: Sex hormones and risk of epilepsy: A bidirectional Mendelian randomization study
Source: Front Mol Neurosci. 2023 Apr 11;16:1153907. doi: 10.3389/fnmol.2023.1153907 (PMC10126428; doi:10.3389/fnmol.2023.1153907)

Supplementary Table 1. Summary data from all GWAS used in current study.

Phenotype	Cases	Controls	Number of SNPs	PMID
testosterone	425,097	-	16,578,494	32042192
testosterone in males	194,553	-	16,577,312	32042192
testosterone in females	230,454	-	16,580,850	32042192
estradiol	50,828	260,847	7,542,442	34255042
estradiol in males	13,367	134,323	7,871,694	34255042
estradiol in females	37,461	126,524	7,870,546	34255042
estradiol in males in replication	206,927	-	16,582,267	32042192
progesterone	2,619	-	n.a.	31169883
progesterone in males	1,358	-	n.a.	31169883
progesterone in females	1,261	-	n.a.	31169883
epilepsy	15,212	29,677	4880492	30531953

SNP, single nucleotide polymorphism; GWAS, genome-wide association study; PMID, PubMed ID; n.a., not available.

Supplementary Table 2. Effect sizes can be detected with the power of 0.8 given the sample size, proportion of cases and variance explained by instrumental variables.

exposure trait	sample size of outcome GWAS	proportion of cases in outcome GWAS	variance explained by instrumental variables	effect size
epilepsy as outcome				
testosterone	44889	0.34	1.62E-02	0.215
testosterone in males	44889	0.34	4.05E-02	0.140
testosterone in females	44889	0.34	4.06E-02	0.140
estradiol	44889	0.34	1.12E-03	0.718
estradiol in males	44889	0.34	3.84E-03	0.419
estradiol in females	44889	0.34	6.64E-04	0.888
estradiol in males in replication	44889	0.34	2.57E-03	0.501
progesterone	44889	0.34	1.54E-02	0.215
epilepsy as exposure				
testosterone	425097	-	6.82E-03	0.095
testosterone in males	194553	-	6.67E-03	0.157
testosterone in females	230454	-	6.22E-03	0.148
estradiol	311675	0.16	7.38E-03	0.148
estradiol in males	147690	0.09	7.38E-03	0.262
estradiol in females	163985	0.23	8.02E-03	0.174

GWAS, genome wide association study.

Supplementary Figure 1. Schematic analysis workflow.

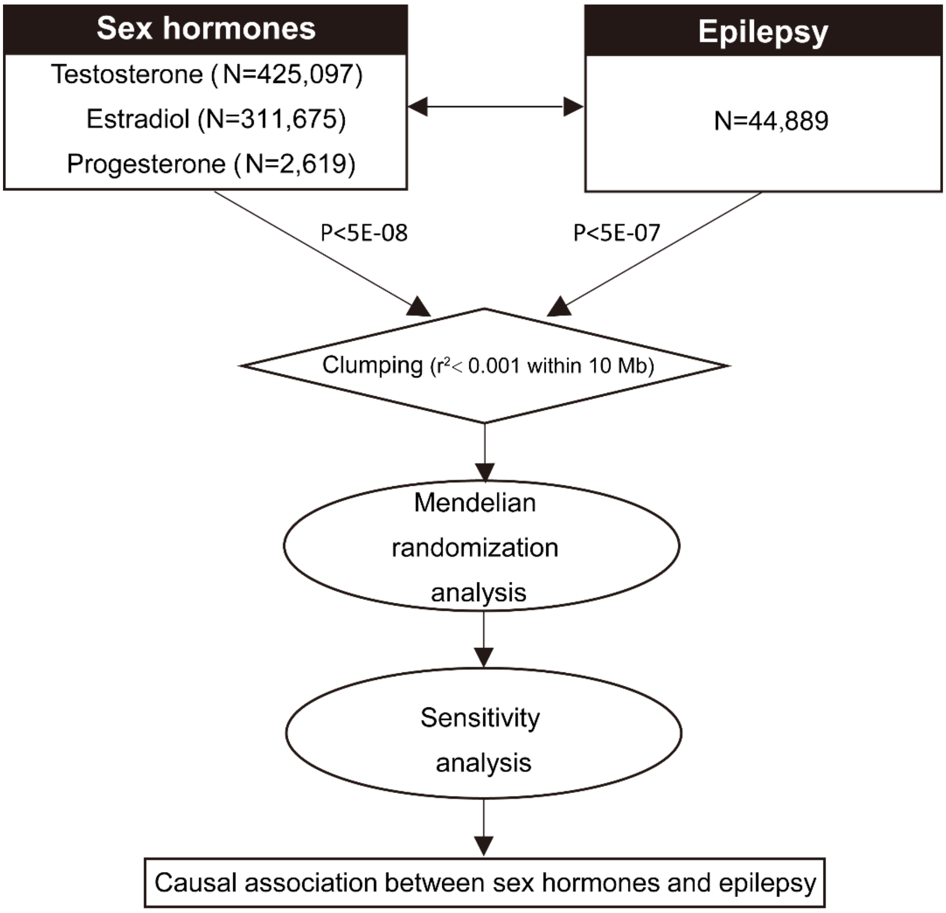

Supplementary Figure 2. Mendelian randomization analysis results for estradiol in females on risk of epilepsy.

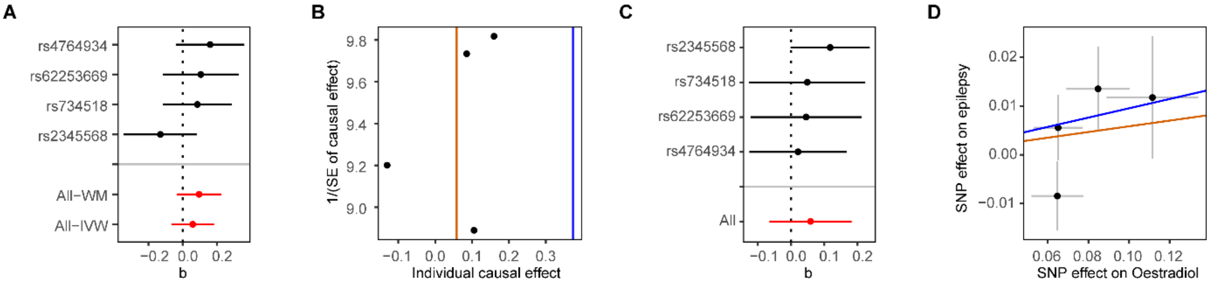

(**A**) Forest plot of the causal effect of estradiol in females on epilepsy. (**B**) Funnel plot showing the estimation using the inverse of the standard error of the causal estimate with each individual SNP as a tool. The vertical line represents the estimated causal effect. SNP, single nucleotide polymorphism. (**C**) Forest plot of the results of the leave-one-out sensitivity analysis, where each SNP was iteratively removed from the instrumental variables. (**D**) Scatter plot of SNP effects on estradiol in females and epilepsy. The 95% CI for the effect size on epilepsy is shown as vertical lines, while the 95% CI for the effect size on estradiol in females is shown as horizontal lines. The slope of fitted lines represents the estimated MR effect per method.

Supplementary Figure 3. Mendelian randomization analysis results for estradiol on risk of epilepsy in both sexes.

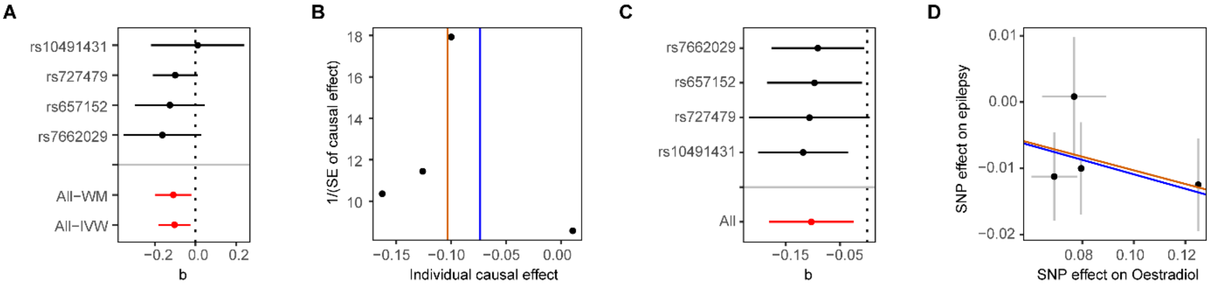

Supplement: Supplementary file 1 [file Data_Sheet_1.PDF]
